# Supplementary figures and images for: Crosstalk between guanosine nucleotides regulates cellular heterogeneity in protein synthesis during nutrient limitation
Source: PLoS Genet. 2022 May 20;18(5):e1009957. doi: 10.1371/journal.pgen.1009957 (PMC9173625; doi:10.1371/journal.pgen.1009957)

# S1 Fig

## S1A

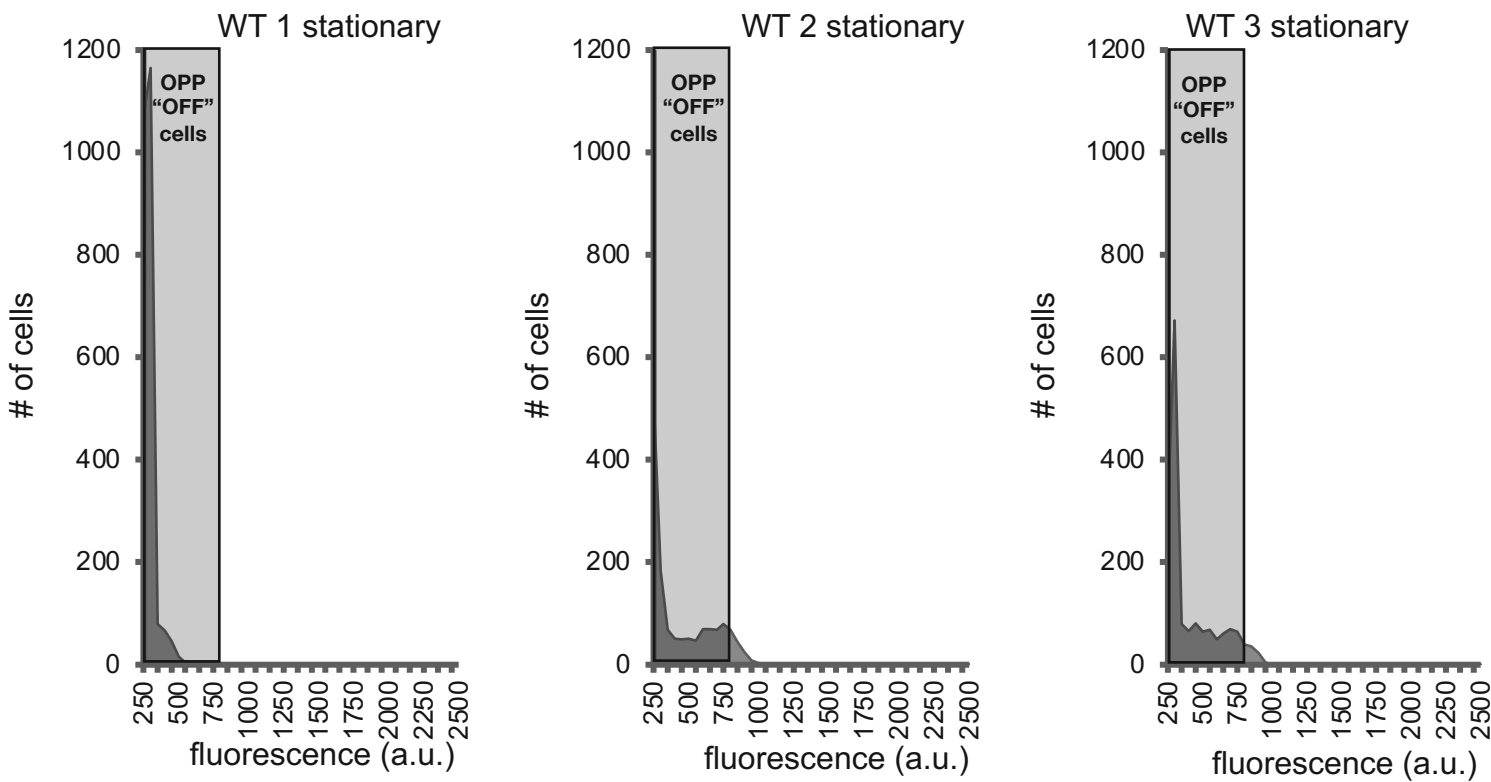

## S1B

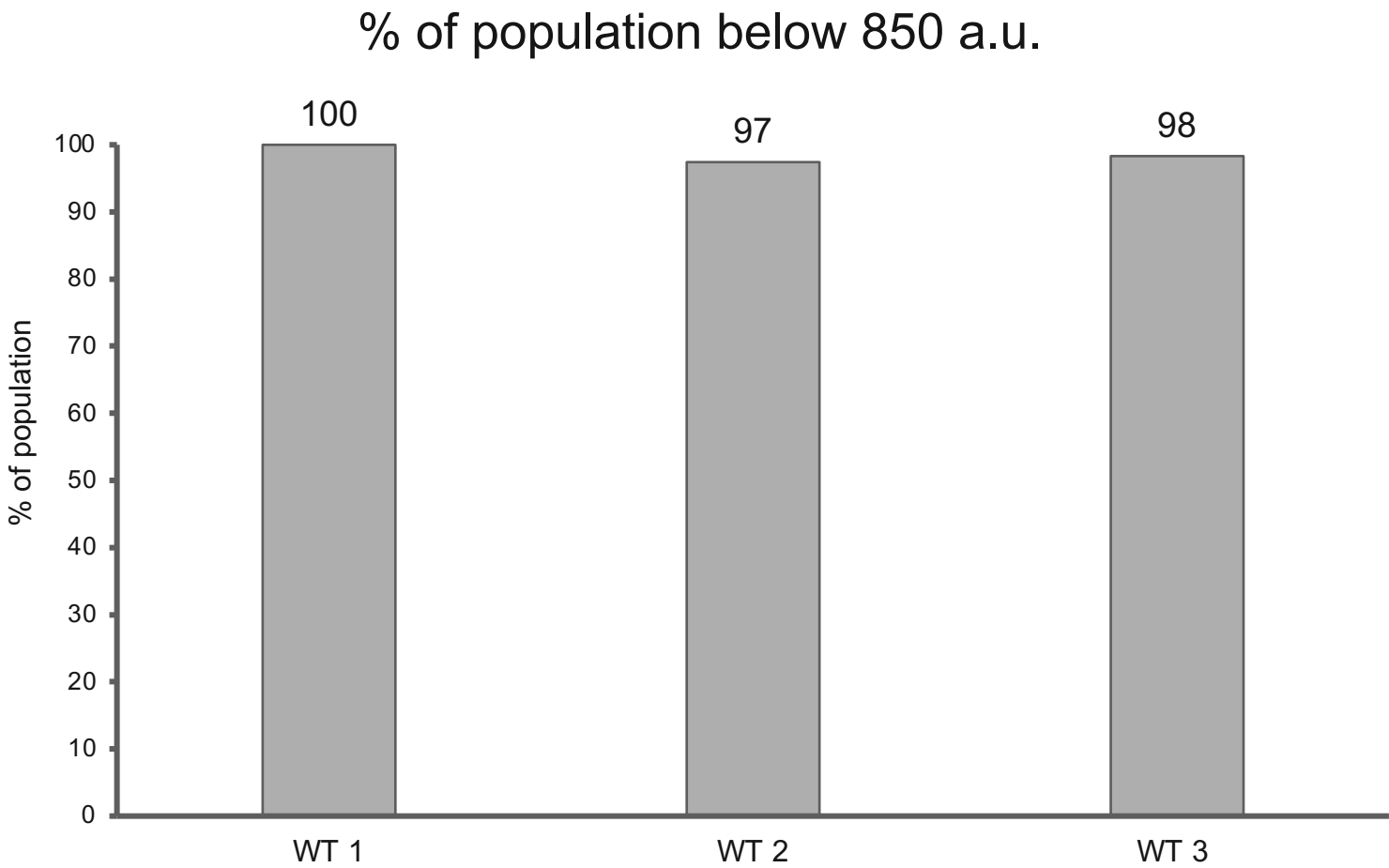

Supplement: S1 Fig — Threshold for OPP “OFF” cells was determined as the fluorescence value (850 a.u.) that is higher than >95% of cells of OPP labeled wildtype B. subtilis during stationary phase across three independent experiments. (A) Three representative distributions of OPP labeled wildtype B. subtilis. Gray box shows cutoff for cells with low rates of protein synthesis (“OFF”). (B) Quantitation of % of population below the threshold determined as “OFF” in the three experiments in A (means ± SDs). (PDF) [file pgen.1009957.s001.pdf]

# S2 Fig

S2A

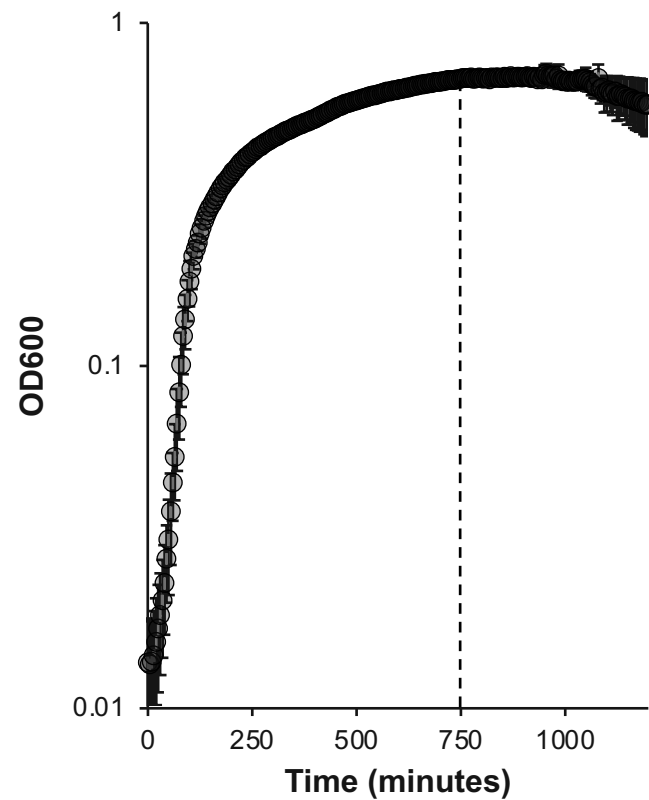

S2B

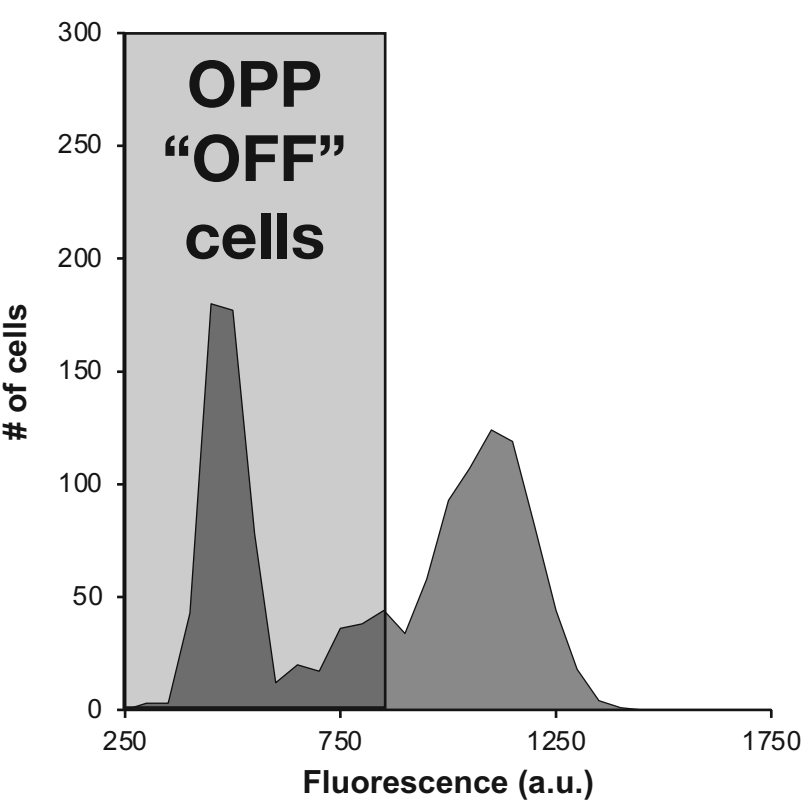

Supplement: S2 Fig — (A) Growth curve of wildtype B. subtilis showing point (OD600 ~0.685) where cells were labeled with OPP (dashed line). (B) Representative distribution of OPP labeled wildtype B. subtilis. Gray box shows cutoff for cells with low rates of protein synthesis (“OFF”). Threshold (850 a.u.) is the value higher than >95% of cells of wildtype B. subtilis labeled with OPP in stationary phase across three independent experiments. (see S1 Fig). (PDF) [file pgen.1009957.s002.pdf]

# S3 Fig

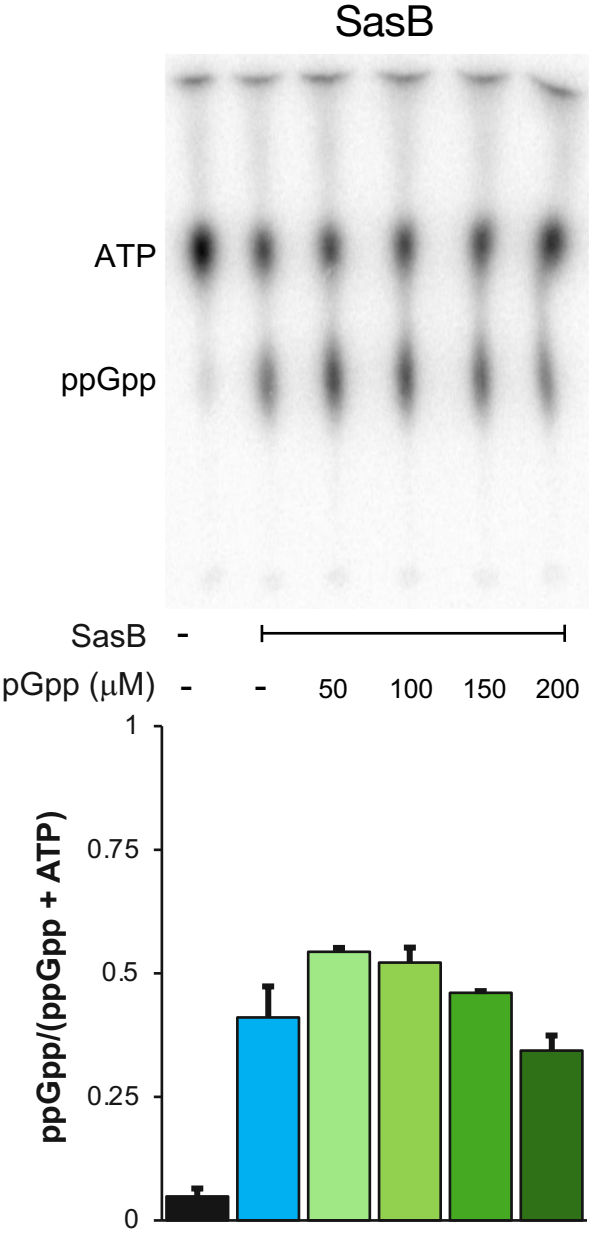

Supplement: S3 Fig — (Top) representative TLC analysis of wildtype SasB activity in the absence of allosteric activation (no pppGpp added) and with increasing concentrations of pGpp (uM). (Bottom) ratio of ppGpp calculated using the formula, ppGpp/ATP + ppGpp. Statistical analysis (t-test) showed no significance (p > 0.05) between any reaction containing SasB whether or not pGpp was included. Statistical analysis was performed on three separate experiments (means ± SDs). (PDF) [file pgen.1009957.s003.pdf]

# S4 Fig

**A**

Wildtype

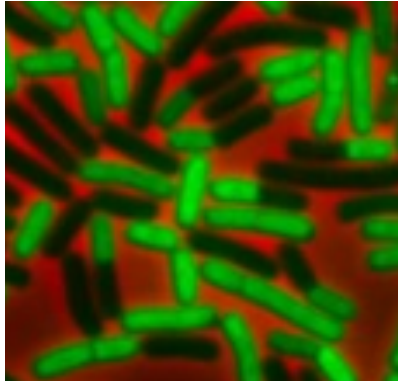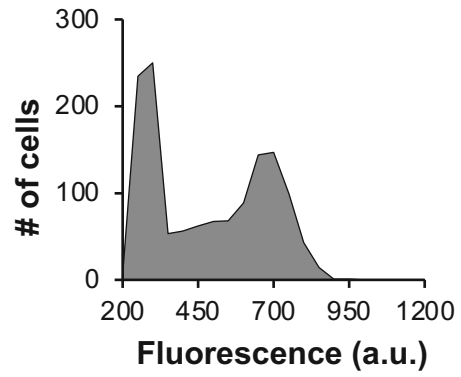

**B**

*ΔnahA*

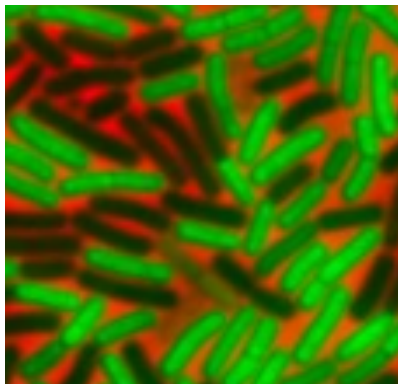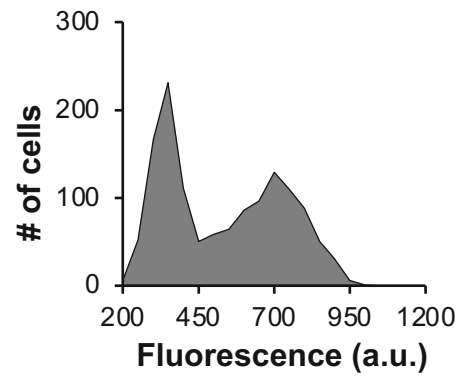

Supplement: S4 Fig — (A, B) Representative pictures and population distributions of OPP labeled (A) wildtype (JDB1772), (B) ΔnahA (JDB4095) strains during late transition phase. (PDF) [file pgen.1009957.s004.pdf]
