# Supplementary material for: Crosstalk between guanosine nucleotides regulates cellular heterogeneity in protein synthesis during nutrient limitation
Source: PLoS Genet. 2022 May 20;18(5):e1009957. doi: 10.1371/journal.pgen.1009957 (PMC9173625; doi:10.1371/journal.pgen.1009957)
Supplement: S1 Table — (PDF) [file pgen.1009957.s005.pdf]

S1 Table. Plasmids used in this study

pETPHOS	Lab stock	
pMINIMAD2	(6)	
AEC 127	(2)	
pETPHOS WT *sasB*	This study	
pETPHOS *sasB*^{F42A}	This study	
pETPHOS *yvcI*	This study	
pMINIMAD2 *relA*^{Y308A}	This study	
pMINIMAD2 *sasB*^{F42A}	This study	
AEC 127 PsasB	This study	
