# Supplementary material for: Crosstalk between guanosine nucleotides regulates cellular heterogeneity in protein synthesis during nutrient limitation
Source: PLoS Genet. 2022 May 20;18(5):e1009957. doi: 10.1371/journal.pgen.1009957 (PMC9173625; doi:10.1371/journal.pgen.1009957)
Supplement: S2 Table — (PDF) [file pgen.1009957.s006.pdf]

S2 Table. Strains used in this study

168 trpC2 (WT)	Lab stock	JDB 1772
trpC2 ΔsasA::kan	(1)	JDB 4310
trpC2 ΔsasB::tet	(1)	JDB 4311
sacA::P_{sasB}*-YFP (cm)*	This study	JDB 4341
sacA::P_{sasA}*-YFP (cm)*	(2)	JDB 4030
trpC2 $sasB^{F42A}$	This study	JDB 4340
trpC2 $relA^{Y308A}$	This study	JDB 4300
trpC2 ΔsasA::kan ΔsasB::tet	(1)	JDB 4312
trpC2 ΔsasA::kan $relA^{Y308A}$	This study	JDB 4301
168 trpC2 Δ*prpC*	(7)	JDB 1773
168 trpC2 Δ*prkC*	(7)	JDB 1774
DH5α pMINIMAD2 $relA^{Y308A}$	This study	JDE 3115
DH5α pMINIMAD2 $sasB^{F42A}$	This study	JDE 3135
DH5α AEC 127 P_{sasB}	This study	
BL21 pETPHOS *WT sasB*	This study	JDE 3136
BL21 pETPHOS $sasB^{F42A}$	This study	JDE 3137
BL21 pETPHOS *yvcI*	This study	JDE 3138
