# Supplementary material for: Crosstalk between guanosine nucleotides regulates cellular heterogeneity in protein synthesis during nutrient limitation
Source: PLoS Genet. 2022 May 20;18(5):e1009957. doi: 10.1371/journal.pgen.1009957 (PMC9173625; doi:10.1371/journal.pgen.1009957)
Supplement: S3 Table — (PDF) [file pgen.1009957.s007.pdf]

S3 Table Oligonucleotides used in this study

GGCTAGAATTCTGATGCTCTTCCTTTCCG	This study	yjbM operon promoter EcoRI F
GGCTAGGATCCACAAAGTACAGATTCATTTT	This study	yjbM operon promoter BamHI R
GGGCCCGAATTCGATGACAAACAATGGGAG	This study	F42A yjbM EcoRI F pMINIMAD2
GGGCCCGTCGACTTGTTGCTCGCTTCCT	This study	F42A yjbM SalI R pMINIMAD2
TTCACCGATCGAAGCTGTGACCGGACGCG	This study	yjbM F42A F
CGCGTCCGGTCACAGCTTCGATCGGTGAA	This study	yjbM F42A R
CATCTTTCGTTTTTTTCTTG	This study	Y308A relA EcoRI F pMINIMAD2
TGGGCTTCATTCGTTTTG	This study	Y308A relA BamHI R pMINIMAD2
AGCCGAATATGGCTCAATCGCTTCA	This study	Y308A relA F
TGAAGCGATTGAGCCATATTCGGCT	This study	Y308A relA R
CCGGGCATATGGATGACAAACAATGG	This study	yjbM NdeI F
GGGAAAACTAGTCTATTGTTGCTCGCTTCC	This study	yjbM SpeI R
GGGCCCCATATGGTGACGTACTTGCAAAGA	This study	yvcI NdeI F
GGGCCCACTAGTCTATTTGATGTGCTGCGG	This study	yvcI SpeI R
