# Supplementary material for: Crosstalk between guanosine nucleotides regulates cellular heterogeneity in protein synthesis during nutrient limitation
Source: PLoS Genet. 2022 May 20;18(5):e1009957. doi: 10.1371/journal.pgen.1009957 (PMC9173625; doi:10.1371/journal.pgen.1009957)
Supplement: Attachment — Submitted filename: Response to Reviewers-3.1.22.pdf [file pgen.1009957.s008.pdf]

Response to Reviews

We greatly appreciate the careful attention paid by both reviewers to our manuscript. The comments they provided and suggestions were helpful in improving the manuscript. We address them in detail, as follows:

Reviewer #1: The paper by Diez et al that describes a possible basis for bimodal heterogeneity in translation levels in Bacillus subtilis cells in stationary phase. I have previously reviewed this manuscript for another journal and was disappointed to learn that few, if any, of the suggestions made by either reviewer were incorporated into this version of the manuscript. I thought these comments were generally constructive and have reiterated the key points from both referees here, because they remain fully pertinent.

Response: We appreciate the frustrations of this reviewer and hope that our responses below justify our decision to not incorporate all of the previous suggestions. We would like to mention that several changes suggested were incorporated, particularly with regards to the potential role of the NahA hydrolase. See Supplementary figure S4 (previously S6)

One key issue is the oversimplification of the model whereby RelA produces pppGpp, SasA produces pGpp and SasB produces ppGpp. Each of these enzymes produces a mixture of products and different authors disagree on what the primary functions are in B. subtilis and closely related organisms. RelA and SasB have both been reported to produce mixtures of pppGpp and ppGpp (PMID: 6111556 ; 26124242). Both SasA and SasB have been reported to produce pGpp (PMID: 26124242 ; PMID: 22950019). Indeed, the primary reference cited in favor of the model that SasA produces pGpp and SasB produces ppGpp (ref 12; PMID: 22950019), was unable to distinguish between pGpp and ppGp by mass-spectrometery and favored the later as the most likely product of SasA in the discussion. This paper was not nearly as categorical as the current authors about which enzyme preferentially produces which product. Lastly, others have shown have shown that although SasA-expressing cells accumulate pGpp, most pGpp is from the conversion of ppGpp to pGpp by the hydrolase NahA (PMID: 32983059 ; 33097692). Ideally the authors should compare the activity of the 3 enzymes side-by-side with GTP, GDP and GMP as substrates. In the absence of such an experiment, the authors should be much more broad-minded about presentation of the model.

Response: We thank the reviewer for their comment, but we respectfully disagree with their interpretation of the literature and its relevance to our study. We discuss this in detail below, but Fig. 6 is not a model, but an effort to integrate the observations of the paper. As such, it necessarily does not account for all of the possible permutations. That is, it is of course formally possible that one of the synthases makes more than one product and that this second product also has an effect on our phenotype. However, we have either demonstrated (Fig 4) or cited appropriate references that show that a specific nucleotide has a biochemical activity that is noted in Fig.6.

The physiological activities of the three (pp)pGpp synthetases in *B. subtilis* have been examined in Wendrich *et al.* (Mol Micro 1997) and in Tagami *et al. (*Micro Open 2012). We rely on these publication because they observe the predominant phosphorylated guanosines following activation of each of the three (pp)pGpp synthases in *B. subtilis* cells and therefore more accurately correspond to the physiological situation examined in our paper. We agree with Reviewer #1 that each of the enzymes in question can make a mixture of products, perhaps not surprising given the similarity of the substrates (GTP, GDP, GMP). However, the available data on the activity of the *B. subtilis* enzymes *in vivo* indicates that each *preferentially* (although not exclusively) produces a particular member of the (pp)pGpp family. Importantly, our interpretations do not depend on whether on not a particular enzyme exclusively produces one product.

Reviewer #1 also lists studies that have shown differing biochemical activities of the (pp)pGpp synthetases than those that we have cited. Briefly we would like to describe why these studies are not relevant to the

interpretation of our experiments. Firstly, reviewer #1 cites (PMID: 6111556) from 1981 that identified the guanosine nucleotides produced by RelA by using a *relA* deletion strain but at that time, it was not known that *B. subtilis* also has accessory (pp)pGpp synthetases. In fact, deletion of the entire *B. subtilis relA* gene is accompanied by compensatory mutations in *sasA* and *sasB*, presumably because the hydrolytic activity of RelA is necessary in a strain with active SasA and SasB. Our study therefore uses a (pp)pGpp^0 strain instead of a single *relA* deletion to analyze RelA activity. The second study that reviewer #1 cites is from a separate Gram positive organism (*E. faecalis*). While *E. faecalis* does have three synthetases with homology to the *B. subtilis* synthases, our work is restricted to (pp)pGpp biology in *B. subtilis.* For example, we observe heterogeneity in *B. subtilis*, but we have not investigated whether this is also the case in *E. faecalis.*

Reviewer #1 suggested that we test the activity of RelA, SasA and SasB *in vitro* with all the potential substrates (GTP, GDP, and GMP). However, the in vitro activity of these enzymes determined using arbitrary concentrations of each enzyme and each substrate will not be as informative as the published data cited in our manuscript concerning the *in vivo* activity of these enzymes. In particular, our central observation of heterogeneity in protein synthesis suggests that there is likely also heterogeneity in levels of substrates such as GTP, GDP, GMP, so correlating any in vitro data with our in vivo genetic observations would be very difficult.

We appreciate the comment about the uncertainty regarding the SasA product and we have added text (lines 65) to our statement about the SasA product, making it less certain. But importantly, our interpretations, largely based on genetic observations, do not depend on whether it makes pGpp or ppGp.

Lastly, to address the importance of NahA on production of pGpp, we added an experiment where we observed heterogeneity in OPP incorporation in a *ΔnahA* strain and found that it was indistinguishable from that occurring in a WT strain. This is likely due to the observed effect of NahA on not only pGpp levels, but also on levels of pppGpp and ppGpp. That is, while SasA presumably affects pGpp levels without changing levels of pppGpp, NahA changes both levels of these nucleotides. This is a key point because our data shows that both of these nucleotides affect SasB activity. We appreciate that under certain conditions as cited by the reviewer, NahA activity may be the central source of pGpp/ppGp but our study is looking at a specific condition (late transition phase) where that appears not be true.

The fluorescence cut-off value used to define cells as 'OFF' for translation was determined as the magnitude of OPP labeling of a stationary phase culture that captures 95% of the population (850 RFU). Unless I am misinterpreting Figure S1, the box showing the 'OFF' population represents <50% of the cells shown in this experiment, not 95% (I cut out the printed graph and weighed the peaks just to be sure). It also seems strange to assume that 95% of WT cells should be 'OFF' in stationary phase, when the study is all about population heterogeneity after all. Is this based on some previous determination? From visual inspection of the profiles shown throughout the paper, it seems that there is always a valley around 700-750 AU ; this might be a better choice for the cut-off.

Response: We apologize for the confusion. The gray box in Figure S1B labeled 'OPP "OFF" cells' is an overlay of the 'OFF' cutoff we determined from *stationary* phase cells as applied to a *transition* phase population. Figure S1 is meant to be a visual representation of how the percentage of 'OFF' cells looks like during late transition phase, which is why the box covers about 50% of the population. To clarify the determination of the cutoff (which is determined from stationary phase cells) and how this cutoff applies to transition phase cells, we have included an extra supplemental figure. The new Figure S1 shows the determination of the cutoff using three independent experiments of stationary phase cells. Figure S2 (previously S1) is a representation of how the cutoff in the new Figure S1 applies to a transition phase culture. We chose *at least* 95% of the population of stationary phase cells as a determinant for the 'OFF' cutoff because it captures the shutoff of protein synthesis that is present in the entire population as cells go from active growth to

stationary phase. We previously showed the transition in OPP incorporation from active growth to stationary phase (Diez et al. PNAS 2020).

Why were quartiles chosen for the analysis in Fig. 2C and D? Wouldn't it have been more logical just to divide into two groups, representing the two peaks? Is quartile 2 significantly different from 3 and 4? If the authors insist onf sticking with quartiles, please show where these fall on the graph using dotted lines. Also, please change the colors in panel 2B. The two shades of green are barely distinguishable.

Response: We have added dotted lines on the graph to designate the position of the quartiles. We have also changed the green coloring so that the two colors are more distinguishable. See Figure 2

Supplementary Figures S2, S3, and S5 should be deleted and the % 'OFF' cells +/- error and asterisks for significant differences be reported directly on the corresponding main figures in the text, to avoid having to go looking for this data in the supplementary section.

Response: We have made the suggested change. See Figures 1, 3, and 5 and Figures legends for these figures. Lines 103-113, 154-159, and 235-240.

Please replace the term 'Ratio of ppGpp' on the Y-axis to ppGpp/(ppGpp+ATP).

Response: We have made the suggested change. See Figure 4 and Figure S3 (previously S4)

Both referees had a problem with the use of the term epistatic towards the end of the results section. For me, sasB is epistatic to sasA and relA is epistatic to sasA, because the sasB phenotype dominates in the double sasAB mutant and the relA308 phenotype dominates in the sasA relA308 double mutant, ie cells are mostly 'ON' in both cases.

Response: We have eliminated "epistatic" terminology and edited the text in that paragraph to clarify our interpretation. See lines 209-217.

Minor points:
L56 Define RSH enzyme

Response: We have edited line 55 to read "Gram-positive bacteria typically encode a single, bi-functional RSH (RelA-SpoT homolog) enzyme capable of both (p)ppGpp synthesis and hydrolysis as well as two additional small alarmone synthases (SAS) that lack hydrolytic activity."

L98 I am not sure how the authors arrive at the conclusion that 'This observation suggests that the SasA product pGpp does not directly inhibit translation as does ppGpp, but rather acts indirectly'. If anything, the phenotype of the sasA mutant could suggest that pGpp directly ACTIVATES translation, at least at face-value.

Response: We have modified the sentence to read: "This observation suggests that the SasA product pGpp does not directly inhibit translation, as does the SasB product ppGpp" which we think is a straightforward interpretation of our observation that ΔsasA strain exhibits reduced translation as compared to ΔsasB. See lines 100-102

L202 IF2 is not the only translation-relevant target for ppGpp. Protein synthesis is also coupled to nutrient

availability through the effect of ppGpp on ribosomal RNA and tRNA synthesis, and on ribosomal assembly factors.

Response: To our knowledge, the studies we assume the reviewer is citing (in B. subtilis) have only been done in the context of amino acid starvation not during transition phase, as we investigate here. But, to clarify, we have edited line 272 to read "The down-regulation of protein synthesis in *B. subtilis* cells experiencing nutrient limitation occurs in part as a result of ppGpp binding and thereby inhibiting IF2."

Reviewer #2: In their interesting new Ms, Diez et al follow up their observation of a bimodal distribution of protein synthesis attenuation in a B. subtilis cell population, mediated e.g. by inhibiting IF2, under conditions when the the (p)ppGpp alarmone synthesis is induced (Diez et al. (2020) PNAS 117:15565). B. subtilis cells encode a bifunctional Rel synthetase, which can synthesize pGpp, ppGpp and pppGpp from GMP, GDP or GTP and ATP. Rel contains also a hydrolase domain, allowing the degradation of these alarmones.

In addition, there are also two small alarmone synthetases RelP (YwaC, SasA) & RelQ (YjbM, SasB) present in B. subtilis. The overlapping and also possibly distinct activities of the three different alarmones synthesized by the three different synthetases were recently investigated by different Labs and specifically for cellular pGpp in B. subtilis it was observed that a hydrolase NahA can generate pGpp from (p)ppGpp (Yang et al. (2020) Nat Comms 11:5388).

Here the authors investigated the possible involvement of the two small synthetases SasA and SasB in influencing the observed heterogeneity of protein synthesis detected by OPP labelling (Diez et al. (2020) PNAS 117:15565) (Fig 1A). Interestingly, when sasA was deleted, the translation was inhibited (Fig 1C) in most cells. However, a much higher portion of translating cells were observed in the population when sasB was deleted (Fig 1B).

The experiments in Fig 2 somehow imply that the cells with higher sasA transcription correlate more with the observation of higher translation. The authors argue that therefore differences in SasA expression might be more important for the observed changes in heterogeneity in that growth phase.

Interestingly a synthetase mutant (relAY308A) of Rel, which is probably in general the major source of (p)ppGpp displayed less bimodal distribution with mostly translating cells (Fig 3C), similar to ΔsasB cells (Fig 1B). And the same was true for a SasB variant F42A (Fig 3B), which is known not to be allosterically activated by pppGpp anymore. This allosteric activation results in higher pppGpp synthesis (Steinchen et al (2015) PNAS 112:13348). Both rel and sasB mutations result in more translating cell, consistent with an expected lower alarmone concentration in these strains.

The authors suggest that an allosteric regulation of SasB activity via SasA could explain these results. Since Tagami et al (2012 MicrobiolOpen 1:115) observe an increase of cellular pGpp, they suggest that the pGpp synthesized by SasA might interfere with SasB synthetase activity.

To test this hypothesis, the purified SasB and SasBF42A and show that raising amounts of pGpp somehow result in lowered synthesis of pppGpp. In the control experiment with SasBF42A this is not visible but the pppGpp synthesis is generally lower (Fig4).

Since both Rel and SasB influence SasA the double mutant ΔsasAΔsasB and ΔsasA rel Y308A should abolish the effect of ΔsasA, which can be confirmed experimentally as observed in Fig 5.

Based on these observations the authors suggest that the synthesis of pGpp by SasA negatively influences the Rel mediated activation of SasB and thereby the level of protein synthesis probably via IF2 in a bimodal manner (Fig6).

Comments

-To really give support to this model (Fig 6), it is probably important to measure the different alarmone levels (pGpp, ppGpp, pppGpp) in some of the presented key B. subtilis strains. Since the authors are able to separate the different populations by Fax, it might even be possible to determine this not only in the whole population but also in the respective sub-populations.

Response: We have carefully considered this experiment. However, the number of cells that we would need to reliably detect the different guanosine species precludes using FACS given the time it would take to sort sufficient cells to detect (pp)pGpp. Specifically, in the literature, (pp)pGpp is measured using ~10 ml of cells, so ~10x9 cells. In our experience using FACS sorting of *B. subtilis* (PMID 31723135), we can sort ~1,000 cells/sec, so this would require on the order of >100 hours, and is therefore not feasible.

-However, it would already help to utilize in addition to the deletion strains of sasA and sasB synthetase defective SasA and SasB variants (e.g. SasAE154V SasBE139V). With such strains it would be easier to conclude that the observed phenotypes of the ΔsasA and/or ΔsasB strains really depend on the synthetase activity of SasA or SasB. And possible protein-protein interactions of these small synthetases, which might also support the observed genetic interaction, would be less perturbed in these strains.

Response: We appreciate the suggestion, but to our knowledge, no enzymatic activity has been associated, or even hypothesized for any SAS protein other than the previously reported synthase activity. Likewise for any protein-protein interactions. We therefore feel that these additional experiments likely would not provide any additional insight beyond what we present with the deletion mutants.

-It is interesting to note that not only Tagami et al observed the in vivo synthesis of pGpp, when SasA was expressed in trans. Fung et al ((2020) Front Mic 11:2083) observed this too. But in addition, they observed a strong pleiotropic effect on the Bacillus physiology and metabolism, which also include the synthesis of other nucleotide second messengers.
Interestingly, Diez et al ((2020) PNAS 117:15565) show themselves in Fig S3 that a SasA induction in trans abolishes the protein synthesis in all the cells of such a population.
I think one could argue that pGpp synthesized by SasA could also directly or indirectly interfere with many cellular processes, which might also include translation.
Therefore, knowing the cellular concentration of pGpp would be important, since it is not clear at what cellular concentration the pGpp could start to influence SasB synthetase activity or the other observed pleiotropic processes.

Response: We agree with the interpretation of the available data and that the cellular concentration of pGpp would be informative in understanding how pGpp affects pleiotropic processes. We have not measured pGpp concentration in bulk cells since that would not accurately capture the presumed heterogeneity in the concentration of pGpp in a particular cell because certain cells might have high levels compared to other cells. So, it would be best to be able to separate the populations of cells and then measure the levels of pGpp. As discussed above, this is not currently feasible.

-Fig 4 The presented in vitro experiments suggest that the pGpp might only diminish the allosteric activation, maybe by somehow competing with pppGpp? Or maybe pGpp competes with substrates?
A more comprehensive analysis and characterization of the SasB enzyme activity and the in vitro influence of pGpp on SasB, would allow to estimate for example determine affinities of pGpp, which would help to understand which cellular concentrations of pGpp have to be reached in vivo.
There seems to be no unit for the amount of pGpp added to the in vitro assay for the SasB activity. What are the concentrations and are they physiologically relevant?

Response: The concentrations of pGpp added are in the μM level. We have edited Figure 4 to reflect this as well as the figure legend. Concentrations of phosphorylated guanosine nucleotides have been measured previously and are known to reach mM levels during amino acid starvation. However, to our knowledge, absolute levels of pGpp have not been determined in *B. subtilis.*

- Fig S6 Both Yang et al and Fung et al observed that NahA is responsible for the generation of more than 80%

of the cellular pGpp, possibly from (p)ppGpp synthesized by SasA. Therefore, it is very likely that in the absence of NahA the cellular pGpp concentration in such a strain is much reduced.
However, in Fig S6 the heterogeneity of protein synthesis of the ΔnahA strain looks like the wildtype strain. This suggests that in the absence of NahA the probably strongly reduced cellular pGpp does not make a difference, and might therefore not being majorly involved in generating the observed wild type like heterogeneity in translation.

Response: Please see our response to reviewer 1, where we argue that NahA activity in the particular physiological situation that we study (late transition phase) may differ from its observed activity in other conditions reported in the cited studies. In addition, when assessing the role of NahA in heterogeneity it is important to note that deletion of NahA affects levels of pGpp, but also levels of pppGpp and ppGpp. This is unlike deletion of SasA, which presumably does not substantially affect levels of pppGpp. In this case, deletion of NahA produces a physiological situation where the molecules that we identified in Figure 4 to activate (pppGpp) and inhibit activation (pGpp) of SasB are both affected. Given that SasB is essential for the heterogeneity in wildtype cells, the effect of ΔnahA deletion on pppGpp and pGpp is perhaps a reason that ΔnahA does not significantly affect heterogeneity in OPP incorporation.

other comments

Fig 2 The promoter activity might somehow correlate with the translated amount of SasA or SasB, however in the paper of Tagami et al they did a Western blot and showed that they were able to detect SasA only in low levels compared to SasB in the transition growth state.

Response: We're not sure we understand the comment. We see lower levels of SasA (panel B, darker green) as compared to SasB (panel A, darker blue) in transition phase, which is consistent with the data in Tagami et al.

l55, Intro RsgA might be a misleading example since in B. Subtilis RsgA does not bind ppGpp as shown in the cited paper (8) (Corrigan et al)
Response: We have removed RsgA from line 54.

line 63-64 – „B. Subtilis RelA primarily generates pppGpp (...)" (11) This reference from Wendrich et al (1997) might not be the best fit, since at that time the two small synthetases were not known yet, and their additional influence could therefore not be considered in this paper at that time.

Response: We agree that the strain in that paper likely has compensatory mutations in SasA and SasB. We chose to keep this reference because it nicely shows the guanosine nucleotides that are produced during RelA activation *via* amino acid starvation. In the absence of evidence that amino acid starvation activates SasA or SasB, we would argue that this data accurately indicates that the predominant species produced by RelA is pppGpp.
