# Supplementary material for: Crosstalk between guanosine nucleotides regulates cellular heterogeneity in protein synthesis during nutrient limitation
Source: PLoS Genet. 2022 May 20;18(5):e1009957. doi: 10.1371/journal.pgen.1009957 (PMC9173625; doi:10.1371/journal.pgen.1009957)
Supplement: Attachment — Submitted filename: DiezReRev.pdf [file pgen.1009957.s009.pdf]

To me the manuscript by Diez et al in its current form does not present enough evidence to support their model. Interestingly and somehow consistent with this, the authors of the manuscript state in their response to Rev 1 "…Fig. 6 is not a model, but an effort to integrate the observations of the paper."
I was happy that the authors corrected the more technical comments and added some clarifications in the text brought up by Rev 1 & 2.

Three more specific comments

Here my responses to some of the comments from the authors to my questions, comments or suggestions (in blue are my first comments, in red the answers from the authors in the "response to reviewer" letter).

(1) -However, it would already help to utilize in addition to the deletion strains of sasA and sasB synthetase defective SasA and SasB variants (e.g. SasAE154V SasBE139V). With such strains it would be easier to conclude that the observed phenotypes of the ΔsasA and/or ΔsasB strains really depend on the synthetase activity of SasA or SasB. And possible protein-protein interactions of these small synthetases, which might also support the observed genetic interaction, would be less perturbed in these strains.

Response: We appreciate the suggestion, but to our knowledge, no enzymatic activity has been associated, or even hypothesized for any SAS protein other than the previously reported synthase activity. Likewise for any protein-protein interactions. We therefore feel that these additional experiments likely would not provide any additional insight beyond what we present with the deletion mutants.

The argument that something like another SAS activity or possible interaction with other proteins / molecules has not been observed or reported, to not do an experiment, which can exclude certain possibilities, does not appeal to me.
Actually, a number of proteins interacting with (pp)pGpp synthetases and modifying their activity has been reported for different organisms. In B. subtilis a certain competence protein could be an example.
Similar observations were reported for other second messenger pathways (e.g. c-di-AMP or c-di-GMP). For these systems and pathways, various more localized regulatory events, which depend on specific protein-protein interaction were observed. The discussed allosteric regulation could actually also be considered to fall in this category.
At the end of their discussion (p25 l313) the authors state: "We find that this heterogeneity is dependent on the RelA product pppGpp, which allosterically activates SasB, and the SasA product pGpp, which antagonizes this activation." By utilizing the suggested active-site point mutants instead of deletions the authors would much more directly test this hypothesis on Rel or SasA "products", central to their manuscript.

(2) Fig 4 The presented in vitro experiments suggest that the pGpp might only diminish the allosteric activation, maybe by somehow competing with pppGpp? Or maybe pGpp competes with substrates?
A more comprehensive analysis and characterization of the SasB enzyme activity and the in vitro influence of pGpp on SasB, would allow to estimate for example determine affinities of pGpp, which would help to understand which cellular concentrations of pGpp have to be reached in vivo.

There seems to be no unit for the amount of pGpp added to the in vitro assay for the SasB activity. What are the concentrations and are they physiologically relevant?

Response: The concentrations of pGpp added are in the µM level. We have edited Figure 4 to reflect this as well as the figure legend. Concentrations of phosphorylated guanosine nucleotides have been measured previously and are known to reach mM levels during amino acid starvation. However, to our knowledge, absolute levels of pGpp have not been determined in B. subtilis.

With the now given amount of pGpp (50-200µM) (in Fig 4) the pppGpp at 12.5 µM and the SasB enzyme at 0.8 µM (in M&M), it appears that an at least 16 times excess of pGpp over pppGpp is necessary to just abolish the allosteric activation of SasB by pppGpp. Is it known whether such an excess of pGpp over pppGpp was observed or is possible in the cell?

(3) Fig 2 The promoter activity might somehow correlate with the translated amount of SasA or SasB, however in the paper of Tagami et al they did a Western blot and showed that they were able to detect SasA only in low levels compared to SasB in the transition growth state.

Response: We're not sure we understand the comment. We see lower levels of SasA (panel B, darker green) as compared to SasB (panel A, darker blue) in transition phase, which is consistent with the data in Tagami et al.

In Fig 2 the authors can observe a relative increase of the transcriptional promoter sasA fusion (p17 l116): “Cells with higher sasA expression (PsasA-yfp) are more likely to have higher levels of protein synthesis than cells with lower sasA expression (Fig 2D).” This is of course consistent with their model.
However, Tagami et al (2012 MicrobiolOpen 1:115) (Fig 2) do not detect an increase of SasA in the late transition phase by Westernblot. They might be wrong or there could be other e.g. posttranslational events independent of raised transcription, which might influence SasA levels or activity, somehow interfering with SasB activity, resulting in the apparent heterogeneity. Maybe, additional translational YFP fusion to SasA and SasB could be better reporters?
