# Supplementary material for: Crosstalk between guanosine nucleotides regulates cellular heterogeneity in protein synthesis during nutrient limitation
Source: PLoS Genet. 2022 May 20;18(5):e1009957. doi: 10.1371/journal.pgen.1009957 (PMC9173625; doi:10.1371/journal.pgen.1009957)
Supplement: Attachment — Submitted filename: response to reviewers.docx [file pgen.1009957.s010.docx]

**Response to reviewers (our answers in green)**

**Reviewer #1:** The revised ms by Diez et al was again disappointing, with little effort to provide any

of the experimental support requested by either reviewer. My main issue still is the

oversimplification of the model (although the authors say that Fig. 6 is not a model, most readers

will regard it as such). Even though the different (pp)pGpp synthetases may generate preferential

products under specific circumstances, they are nonetheless mixed at best, and in some cases not

yet completely determined. Despite a minor text modification, IF2 comes across as the only

translation target of (p)ppGpp, when several other components of the translational apparatus,

including rRNA and tRNA synthesis, are well known to be (p)ppGpp targets. The paper globally, and

the schematic in Fig. 6, need be redone to incorporate other possibilities. For example, when a

mixture of products is known to be produced, the favored one could have a larger font in Fig. 6.

pGpp could have an asterisk to indicate that the inhibitory effect was shown directly, but ppGp

should also be shown. rRNA and tRNA should be mentioned along with IF2 as targets of (pp)pGpp.

The product of SasA is either pGpp or ppGp, but more likely ppGp, according to the authors of the

cited paper PMID 2950019. For reasons that are unclear, the authors propose to call this mixture

pGpp (which I find a little disingenuous), and then only test pGpp (and not ppGp) in Fig. 4 as a

competitive inhibitor of the allosteric activation of SasB by pppGpp. The mixture should be referred

to as pGpp/ppGp.

We appreciated this perspective and have now eliminated Fig.6 so that there is no confusion. We have modified the text to make clear that IF2 is a direct target of ppGpp; to our knowledge, in B. subtilis, IF2 is the only protein involved in translation has been demonstrated to be an authentic direct (binding) target in vivo. In the paper cited (we assume that the correct PMID is 22950019), the authors state that is "likely that" it is ppGp, but to our understanding there is no explicit data on this point. The most recent report of SasA function (Fung et al 2020) refers to one of its products as 'pGpp' so we have chosen to follow this convention.

As referee 2 pointed out, both Yang et al and Fung et al observed that NahA is responsible for the

generation of more than 80% of the cellular pGpp, possibly from (p)ppGpp synthesized by SasA.

Therefore, it is very likely that in the absence of NahA the cellular pGpp concentration in such a

strain is much reduced and the fact the nahA deletion strain behaves like the wt is not coherent

with the model presented. To explain this discrepancy, the authors make the not very convincing

suggestion that this may not be the case in late transition phase, but do not provide any evidence

to support this. In the absence of experimental evidence, this for me is a potential symptom of an

over-simplified model.

Our conditions (LB, a defined point in late transition phase, Fig S2) are different from those in Yang, where the nahA deletion strain is assessed in "stationary phase" in S7. In addition, there are changes in the nahA strain apart from less pGpp, such as significant increases in pppGpp and ppGpp (Yang et a., 2020, Fig. 3). So, these changes may offset the effect of pGpp, especially since pppGpp is an allosteric activator of SasB. Finally, we note that the Fung et al 2020 paper reports that ~25% of the pGpp generated by overexpression of SasA is not dependent on nahA.

I agree with referee 2 that it is important to measure the different nucleotides present in the

different mutants under the experimental conditions tested to provide support for the model, even

if it is only technically feasible at the population level.

While we appreciate this suggestion, these observations will not yield any insight into the phenomenon of heterogeneity, which the central focus of our manuscript. For example, if we were to measure protein synthesis in bulk, then most of experiments would observe no difference between the wildtype and the various mutants, which is clearly not what we see when we monitor single cells

Minor points:

Fig. S1 should be described in the text. The sentences provided L88-91 reference Fig. S2 and still

refer to 95% of entire population in late transition phase, when this is clearly not the case.

Corrected. See 98-102

It is still not clear to me how the quartiles were designated in Figure 2. Only two seem to be

delineated in Figure 2A and B. Labelling the quartiles would help. Is only the late transition peak

considered? If so how was the growth phase of the two peaks determined?

Corrected

Abstract (L29) There is one ‘p’ too many in (pp)ppGpp

Corrected.

**Review 2**

To me the manuscript by Diez et al in its current form does not present enough evidence to

support their model. Interestingly and somehow consistent with this, the authors of the

manuscript state in their response to Rev 1 “…Fig. 6 is not a model, but an effort to integrate

the observations of the paper.”

I was happy that the authors corrected the more technical comments and added some

clarifications in the text brought up by Rev 1 & 2.

Three more specific comments

Here my responses to some of the comments from the authors to my questions, comments or

suggestions (in blue are my first comments, in red the answers from the authors in the

“response to reviewer” letter).

(1) -However, it would already help to utilize in addition to the deletion strains of sasA and

sasB synthetase defective SasA and SasB variants (e.g. SasAE154V SasBE139V). With such

strains it would be easier to conclude that the observed phenotypes of the ΔsasA and/or

ΔsasB strains really depend on the synthetase activity of SasA or SasB. And possible proteinprotein

interactions of these small synthetases, which might also support the observed genetic

interaction, would be less perturbed in these strains.

Response: We appreciate the suggestion, but to our knowledge, no enzymatic activity has

been associated, or even hypothesized for any SAS protein other than the previously reported

synthase activity. Likewise for any protein-protein interactions. We therefore feel that these

additional experiments likely would not provide any additional insight beyond what we

present with the deletion mutants.

The argument that something like another SAS activity or possible interaction with other

proteins / molecules has not been observed or reported, to not do an experiment, which can

exclude certain possibilities, does not appeal to me.

Actually, a number of proteins interacting with (pp)pGpp synthetases and modifying their

activity has been reported for different organisms. In B. subtilis a certain competence protein

could be an example.

Similar observations were reported for other second messenger pathways (e.g. c-di-AMP or

c-di-GMP). For these systems and pathways, various more localized regulatory events, which

depend on specific protein-protein interaction were observed. The discussed allosteric

regulation could actually also be considered to fall in this category.

At the end of their discussion (p25 l313) the authors state: “We find that this heterogeneity is

dependent on the RelA product pppGpp, which allosterically activates SasB, and the SasA

product pGpp, which antagonizes this activation.” By utilizing the suggested active-site point

mutants instead of deletions the authors would much more directly test this hypothesis on Rel

or SasA “products”, central to their manuscript.

We assume that the reviewer is referring to a study from the Dubnau laboratory (PMID: 25899641) that reports a functionally important interaction between RelA and the ComGA competence protein. In our work, we use a *relA^Y308A^* mutation, which allows us to be certain that effects of this mutation are due to its ability to synthesize (pp)pGpp.

(2) Fig 4 The presented in vitro experiments suggest that the pGpp might only diminish the

allosteric activation, maybe by somehow competing with pppGpp? Or maybe pGpp competes

with substrates?

A more comprehensive analysis and characterization of the SasB enzyme activity and the in

vitro influence of pGpp on SasB, would allow to estimate for example determine affinities of

pGpp, which would help to understand which cellular concentrations of pGpp have to be

reached in vivo.

There seems to be no unit for the amount of pGpp added to the in vitro assay for the SasB

activity. What are the concentrations and are they physiologically relevant?

Response: The concentrations of pGpp added are in the μM level. We have edited Figure 4 to

reflect this as well as the figure legend. Concentrations of phosphorylated guanosine

nucleotides have been measured previously and are known to reach mM levels during amino

acid starvation. However, to our knowledge, absolute levels of pGpp have not been

determined in B. subtilis.

With the now given amount of pGpp (50-200μM) (in Fig 4) the pppGpp at 12.5 μM and the

SasB enzyme at 0.8 μM (in M&M), it appears that an at least 16 times excess of pGpp over

pppGpp is necessary to just abolish the allosteric activation of SasB by pppGpp. Is it known

whether such an excess of pGpp over pppGpp was observed or is possible in the cell?

We do not know if that is the case, especially given that there may very well be heterogeneity in single cells with regard to the activity of SasA.

(3) Fig 2 The promoter activity might somehow correlate with the translated amount of SasA

or SasB, however in the paper of Tagami et al they did a Western blot and showed that they

were able to detect SasA only in low levels compared to SasB in the transition growth state.

Response: We're not sure we understand the comment. We see lower levels of SasA (panel B,

darker green) as compared to SasB (panel A, darker blue) in transition phase, which is

consistent with the data in Tagami et al.

In Fig 2 the authors can observe a relative increase of the transcriptional promoter sasA

fusion (p17 l116): “Cells with higher sasA expression (PsasA-yfp) are more likely to have

higher levels of protein synthesis than cells with lower sasA expression (Fig 2D).” This is of

course consistent with their model.

However, Tagami et al (2012 MicrobiolOpen 1:115) (Fig 2) do not detect an increase of SasA

in the late transition phase by Westernblot. They might be wrong or there could be other e.g.

posttranslational events independent of raised transcription, which might influence Sas

levels or activity, somehow interfering with SasB activity, resulting in the apparent

heterogeneity. Maybe, additional translational YFP fusion to SasA and SasB could be better

reporters?

We don't understand the discrepancy between our data which clearly show higher levels of *sasA* expression in late transition as compared to exponential phase (Fig. 2B) and Tagami, et al., 2012. One possible explanation is that our study and the Tagami study examined *sasA* expression at different points of the growth curve (compare Fig 2A of Tagami with our Fig. S2).
